# The Forefront of Dengue Control

**DOI:** 10.1016/j.bjid.2026.104611

**Published:** 2026-01-13

**Authors:** Luciano Z. Goldani

**Affiliations:** Infectious Diseases Unit, Hospital de Clínicas de Porto Alegre, Universidade Federal do Rio Grande do Sul

Dengue is the most rapidly spreading mosquito-borne viral disease in the world, with no specific antiviral available and remarkable morbidity and mortality. Many tropical and subtropical countries face recurrent epidemics, accounting for a significant burden to health systems through increased hospitalizations, intensified surveillance demands, and the diversion of resources from other priority diseases. In this context, Brazil has developed multi-faceted interventions to combat Dengue including the development of a nationally produced dengue vaccine, and the large-scale deployment and production of biologically modified *Aedes aegypti* mosquitoes. In addition, Brazil has established a dengue genomic surveillance, predictive modeling, and diagnostic innovation, integrating them into a robust public health framework. A recent study provided key genomic insights into chikungunya and dengue virus circulation in Minas Gerais, one of Brazil’s most affected states in 2023, through the generation of 80 CHIKV and 151 DENV-1 genomes^1^. The integration of genomic surveillance with epidemiological genomic monitoring is fundamental to characterizing viral dissemination dynamics and identifying priority areas for targeted control interventions.

Currently, there is only one vaccine recommended by WHO available for dengue prevention. The Q-denga® (TAK-003), a tetravalent attenuated vaccine developed by Takeda Pharmaceuticals in Japan, is recommended in settings with high intensity of dengue transmission and the vaccination course consists of two injections given 3 months apart. The development and a recently ANVISA (Brazilian Health Regulatory Agency) approval of a tetravalent live-attenuated dengue vaccine (Butantan-DV) represents an important achievement in Brazilian biomedical science. Originating from a platform developed by the U.S. National Institutes of Health (NIH), the vaccine was optimized and clinically advanced in Brazil. The vaccine was designed as a single-dose regimen, and provides balanced protection against all four dengue serotypes.

Phase 2 and 3 trials in Brazil have demonstrated robust immunogenicity and a favorable safety profile across age groups, including children and adults^2^^,^ ^3^. Overall, 2-year vaccine efficacy of 79.6 % against symptomatic virologically confirmed dengue has been shown irrespective of baseline serostatus, addressing a critical limitation of earlier vaccines that raised safety concerns for seronegative individuals^4^. Vaccine efficacy against severe dengue or dengue with warning signs was estimated at 89.0% with an average of 3.7 years of follow-up, independent of hospitalization status^5^. The single-dose schedule offers a major operational advantage for public health programs, particularly in resource-limited settings.

Beyond its clinical promise, the Butantan-DV vaccine could offer an integration into the Brazilian National Immunization Program (PNI) that could substantially alter national dengue epidemiology and serve as a model for other endemic regions.

A second relevant contribution was the implementation of *Wolbachia*-based biocontrol. The *Wolbachia* bacterium, when introduced into *Aedes aegypti* populations, reduces the mosquitoes’ ability to transmit dengue, Zika, chikungunya, and yellow fever viruses^6^. It spreads naturally via cytoplasmic incompatibility, and represents a cleaner and more sustainable alternative to traditional insecticide use, avoiding chemical residues and reducing selective pressure for insecticide resistance.

Through partnerships like the World Mosquito Program, Brazil has executed large-scale deployments of *Wolbachia*-infected *Aedes aegypti* in cities such as Rio de Janeiro, Niterói, and Belo Horizonte^7^. Brazilian field studies have reported significant reductions in dengue incidence exceeding 60% in some areas following local *Wolbachia* establishment^8^. A recent study conducted in Campo Grande, a dengue-endemic urban center in Brazil with nearly 900,000 inhabitants, this intervention demonstrated a relevant impact on dengue transmission^9^. Between December 2020 and December 2023, more than 100 million of mosquitoes carrying the wMel strain **of** Wolbachia were released, resulting in high levels of *Wolbachia* introgression across the city. By 2024, the mean *Wolbachia* prevalence reached 86.4%, with 89% of areas achieving stable prevalence ≥60%. In fully treated areas, with a *Wolbachia* prevalence ≥60%), dengue incidence dropped by 63.2% compared to the pre-intervention period. The recently opened biofactory in Curitiba has scaled Brazil’s production capacity, establishing the country as the global leader in supplying Aedes aegypti mosquitoes infected with the Wolbachia bacterium^10^. With the new biofactory, production will reach 100 million of *Wolbachia*-infected eggs per week**.** The program’s coverage, which previously benefited approximately 5 million people, is expected to expand to 140 million individuals across around 40 municipalities in Brazil that have recorded the highest disease incidence in recent years.

Complementing these approaches, sterile insect techniques has been implemented to suppress mosquito populations with fully automated adult mosquito release system operated from an uncrewed aerial vehicle^11^^,^ ^12^. Drone-based release of sterile males represents a significant advance, especially considering the inherently low dispersal range of Aedes mosquitoes. These methods involve releasing male mosquitoes rendered sterile through irradiation, genetic modification, or bacterial incompatibility; their mating with wild females produces no viable offspring. By decreasing the number of competent Aedes mosquitoes, these approaches limit viral circulation in the community and ultimately contribute to a decline in dengue incidence.

In conclusion, Brazilian research institutions, in cooperation with international collaborators, have conducted relevant studies demonstrating that sustained releases of innovative modified mosquitoes can achieve significant local reductions in vector populations and the associated risk of arbovirus transmission. These techniques offer high species specificity and minimal ecological disruption, making them valuable components of integrated vector management. Importantly, when combined with dengue vaccination strategies aimed at reducing human susceptibility such interventions act synergistically to interrupt transmission dynamics. This integrated approach at comparatively low cost offers a promising pathway to substantially reduce the health, social, and economic burden of dengue, not only in endemic low- and middle-income countries but also in regions facing emerging or re-emerging arboviral threats.

## Conflicts of interest

The author declares no conflicts of interest regarding this manuscript.

## References

[1] de Jesus A.C.P., Fonseca P.L.C., Alves H.J. (2024). Retrospective epidemiologic and genomic surveillance of arboviruses in 2023 in Brazil reveals high co-circulation of chikungunya and dengue viruses. BMC Med.

[2] Kallás E.G., Precioso A.P., Palacio R. (2020). Safety and immunogenicity of the tetravalent, live attenuated dengue vaccine Butantan-DV in adults in Brazil: a two-step, double-blind, randomised placebo-controlled phase 2 trial. Lancet Infect Dis.

[3] Kallás E.G., Cintra M.A.T., Moreira J.A. (2024). Live, attenuated, tetravalent Butantan-Dengue vaccine in children and adults. N Engl J Med.

[4] Sridhar S., Luedtke A., Langevin E. (2018). Effect of dengue serostatus on dengue vaccine safety and efficacy. N Engl J Med.

[5] Nogueira M.L., Cintra M.A.T., Moreira J.A. (2024). Efficacy and safety of Butantan-DV in participants aged 2–59 years through an extended follow-up: results from a double-blind, randomised, placebo-controlled, phase 3, multicentre trial in Brazil. Lancet Infect Dis.

[6] Hoffmann A.A., Montgomery B.L., Popovici J. (2011). Successful establishment of *Wolbachia* in *Aedes* populations to suppress dengue transmission. Nature.

[7] Garcia G.A., Sylvestre G., Aguiar R. (2019). Matching the genetics of released and local *Aedes aegypti* populations is critical to assure *Wolbachia* invasion. PLoS Negl Trop Dis.

[8] Pinto S.B., Riback T.I.S., Sylvestre G. (2021). Effectiveness of *Wolbachia*-infected mosquito deployments in reducing the incidence of dengue and *Aedes*-borne diseases in Niterói, Brazil: a quasi-experimental study. PLoS Negl Trop Dis.

[9] FdM Batista, PM Carcamo, Nelson E. (2026). The impact of large-scale release of *Wolbachia* mosquitoes on dengue incidence in Campo Grande, Brazil: an ecological study. **Lancet Reg Health Am**.

[10] Ministério da Saúde (BR) (2025 Jul 19). https://www.gov.br/saude/pt-br/assuntos/noticias/2025/julho/ministerio-da-saude-inaugura-a-maior-biofabrica-de-wolbachia-do-mundo.

[11] Carvalho D.O., McKemey A.R., Garziera L. (2015). Suppression of a field population of *Aedes aegypti* in Brazil by sustained release of transgenic male mosquitoes. PLoS Negl Trop Dis.

[12] Bouyer J., Culbert N.J., Dicko A.H., Go M. (2020). Field performance of sterile male mosquitoes released from an uncrewed aerial vehicle. Sci Robot.

